# The Jews: A Study of Race and Environment

**Published:** 1911-09

**Authors:** 


**The Jews : A Study of Race and Environment.** By MAURICE FISHBERG. London : The Walter Scott Publishing Co 1911.

This book is the product of much erudition and thought. The author being himself a Jew, and dealing with his own race, and that the most interesting one, anthropologically and socially, in the history of the world, has naturally the most intense interest in his subject. Moreover, he has a formed opinion on most of the vexed questions therein included, but scrupulously endeavours to be fair, and is careful to produce the evidence against his own view well and distinctly.

The range of his work is very wide, dealing not only with the anthropology and pathology of the Jews, but largely with their history and sociology and the probable future trend of most matters Jewish.

Briefly, his views run counter to the ordinary opinions as to the purity of breed and the permanently differentiated character, physical and moral, of this extraordinary race. He points out that a Jew may be tall or short, blond or brunet, long- or broad-headed ; that even the supposed characteristic nose occurs in quite a minority of the people ; that in all these respects the Jews seem to approximate to whatever breed of Gentiles they have lived among ; and that there is in many cases plenty of evidence of the occurrence of exogamy, and of the conversion and incorporation of considerable numbers of Gentiles. He accordingly ascribes the supposed remarkable capacity for acclimatisation to the fact that there are many Jewish types, not one only.

His chapters on pathological characteristics will naturally be interesting to his medical readers.

He discusses the generally known and recognised comparative immunity of Jews from phthisis, which he thinks must be due chiefly to their having become immune through their having been huddled together in Ghettos or Jewries for so many generations, during which those whose constitutions could not adapt themselves to these horrible conditions must have died out. In this connection he quotes the mortality statistics of the Ghetto of Vienna in 1648–69, when nearly one-third of the deaths were attributed to diseases probably tuberculous. He notes also that immigrants into the United States from the peasantry of Eastern Europe have an "actually appalling" mortality from phthisis when they settle in crowded American cities, while their Jewish neighbours escape, or at worst contract the disease in a slower and more tractable form.

Of diseases or conditions which prevail more among Jews than Gentiles, Fishberg acknowledges several. Such are short-sight and colour-blindness, both probably due to the fact that the Jews are hereditary town-dwellers. Affections of the nervous system are rife among them, as one might expect from the anxious nature of the lives that most of them lead. Epilepsy may be an exception, owing probably to the fact that Jews, so long as they adhere to the dictates of their religion, are comparatively exempt from syphilis and alcoholism. But idiocy, some forms of insanity, hysteria and deafmutism are all confessedly more prevalent, even to a marked degree. As predisposing causes, the over-anxious lives they lead, over-use of the brain and too little of the muscles, and the frequent marriage of near kindred, seem to supply a sufficient explanation. Suicide used to be rare, but its frequency is rapidly increasing under what Morselli calls the "universal and complex influence to which we give the name 'civilisation'"; and this is the case both in America and in Western Europe. Diabetes is well known to be particularly rife among Jews, and that not only among the rich but, though to a somewhat less extent, also among the poor and miserable.

During a great part of the last century the Jewish population in most countries appeared to be rapidly increasing, not so much from the high proportion of births as from the small one of deaths, the result in the main of a higher standard of morality and of their avoidance of dangerous occupations. It seemed as though the future of the world was in the lap of the Jews, who not only increased in number but also in wealth, and that to such a degree that they were likely to engross all the wealth of the world and with that the power. But our author shows that the unexpected is happening. The Jew is dropping his religion and intermarrying with Gentiles: moreover the enormous increase of his wealth is having its usual effect in lessening fertility; and in Western Europe and America, as is the case in prosperous France, the birth-rate is actually falling below the rate of mortality.

